# Influenza Virus Polymerase Mutation Stabilizes a Foreign Gene Inserted into the Virus Genome by Enhancing the Transcription/Replication Efficiency of the Modified Segment

**DOI:** 10.1128/mBio.01794-19

**Published:** 2019-10-01

**Authors:** Yuri Furusawa, Shinya Yamada, Tiago Jose da Silva Lopes, Jayeeta Dutta, Zenab Khan, Divya Kriti, Harm van Bakel, Yoshihiro Kawaoka

**Affiliations:** a Division of Virology, Department of Microbiology and Immunology, Institute of Medical Science, University of Tokyo, Tokyo, Japan; b Department of Pathobiological Sciences, School of Veterinary Medicine, University of Wisconsin—Madison, Madison, Wisconsin, USA; c Department of Genetics and Genomic Sciences, Icahn School of Medicine at Mount Sinai, New York, New York, USA; d Department of Special Pathogens, International Research Center for Infectious Diseases, Institute of Medical Science, University of Tokyo, Tokyo, Japan; University of Hong Kong

**Keywords:** foreign gene insertion, genetic stability, influenza, recombinant virus, virus polymerase

## Abstract

We previously attempted to establish a reporter influenza virus by inserting the gene for the Venus fluorescent protein into the NS segment of influenza A/Puerto Rico/8/34 (PR8, H1N1) virus to yield WT-Venus-PR8. Although the inserted Venus gene was deleted during serial passages of WT-Venus-PR8, we discovered that the PB2-E712D mutation stabilizes the Venus gene. Here, we explored the mechanisms by which Venus gene deletion occurs and how the polymerase mutation stabilizes the Venus gene. Deep sequencing analysis revealed that PB2-E712D does not cause an appreciable change in the mutation rate, suggesting that the stability of the Venus gene is not affected by polymerase fidelity. We found by using quantitative real-time PCR that WT-Venus-PR8 induces high-level interferon beta (IFN-β) expression. The induction of IFN-β expression seemed to result from the reduced transcription/replication efficiency of the modified NS segment in WT-Venus-PR8. In contrast, the transcription/replication efficiency of the modified NS segment was enhanced by the PB2-E712D mutation. Loss of the Venus gene in WT-Venus-PR8 appeared to be caused by internal deletions in the NS segment. Moreover, to further our understanding of the Venus stabilization mechanisms, we identified additional amino acid mutations in the virus polymerase complex that stabilize the Venus gene. We found that some of these amino acids are located near the template exit or the product exit of the viral polymerase, suggesting that these amino acids contribute to the stability of the Venus gene by affecting the binding affinity between the polymerase complex and the RNA template and product.

## Abstract (precis)

The reverse genetics method of influenza virus generation has enabled us to generate recombinant viruses bearing modified viral proteins. Recombinant influenza viruses expressing foreign genes have become useful tools in basic research, and such viruses can be utilized as efficient virus vectors or multivalent vaccines. However, the insertion of a foreign gene into the influenza virus genome often impairs virus replication, and the inserted genes are unstable. Elucidation of the mechanisms of foreign gene stabilization will help us to establish useful recombinant influenza viruses.

## INTRODUCTION

Influenza A viruses are segmented negative-strand RNA viruses that belong to the family *Orthomyxoviridae*. The genome of influenza A virus consists of eight RNA segments encoding different viral proteins. With the development of reverse genetics for influenza viruses (1), it became possible to generate recombinant viruses bearing modified viral proteins. Recombinant viruses expressing foreign proteins such as reporter proteins are now useful tools in basic research. Moreover, influenza viruses stably expressing foreign genes may be used as virus vectors or multivalent vaccines. For example, an influenza virus expressing a modified hemagglutinin (HA) segment, in which polypeptides of the Bacillus anthracis protective antigen were inserted, expresses chimeric HA proteins stably and induces antibody responses against both the HA and B. anthracis antigens (2). In another study, insertion of the human interleukin-2 gene into the influenza NS segment enhanced the CD8^+^ immune response to viral antigens (3). However, insertion of foreign genes into virus genomes often impairs virus replication (4, 5), and inserted sequences are not stable during the replication cycle (6).

Previously, we attempted to establish a reporter influenza virus that would allow us to visualize virus-infected cells as a tool to understand influenza virus-induced pathology (7). The gene of the Venus fluorescent protein was inserted into the NS segment of influenza A/Puerto Rico/8/34 (PR8, H1N1) virus to yield WT-Venus-PR8. However, WT-Venus-PR8 was significantly attenuated, and the inserted Venus gene was deleted during serial virus passages. We found that an E-to-D mutation at position 712 of the polymerase subunit PB2 (PB2-E712D) stabilized the inserted Venus gene (7, 8). Furthermore, we also established H5N1 virus carrying the Venus gene, which was inserted into the NS segment from PR8 (Venus-H5N1) (7). Although, like WT-Venus-PR8, WT-Venus-H5N1 showed moderate virulence and low Venus expression, we acquired a variant that became more lethal to mice and stably expressed Venus after mouse adaptation. We found that a V-to-A mutation at position 25 of the polymerase subunit PB2 and a R-to-K mutation at position 443 of the polymerase subunit PA contributed to the stable maintenance of the Venus gene (9). These results indicate that the composition of the viral polymerase plays an important role in the stabilization of the inserted foreign gene. However, the mechanisms by which the Venus gene can be deleted and how polymerase mutations stabilize the Venus gene have remained unknown.

In this study, we explored the mechanisms of Venus gene stabilization by comparing events upon infection with WT-Venus-PR8 and Venus-PR8 possessing the PB2-E712D mutation (Venus-PR8-PB2-E712D). We examined polymerase fidelity and RNA and protein expression in infected cells, and we performed sequencing analysis coupled with coinfection experiments to determine how the Venus gene is deleted. Moreover, we identified additional mutations that contribute to the stabilization of the Venus gene to further our understanding of the stabilization mechanisms.

## RESULTS

### Loss of Venus expression in WT-Venus-PR8 restores replication efficiency.

**FIG 1:**
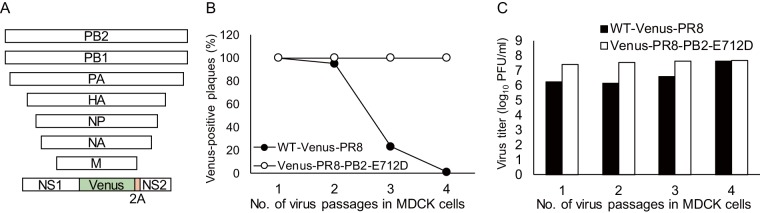
(A) Schematic structure of the eight viral RNA segments contained in WT-Venus-PR8. 2A, protease 2A autoproteolytic site. (B) Each virus was passaged in MDCK cells. The proportion of Venus-expressing plaques in virus stocks from different passages was determined in MDCK cells by using fluorescence microscopy. (C) The virus stocks from different passages were titrated by use of plaque assays in MDCK cells.

We prepared WT-Venus-PR8 and Venus-PR8-PB2-E712D by using reverse genetics as previously described (1). The gene of the Venus fluorescent protein was inserted into the NS segment as illustrated in Fig. 1A (7). First, we confirmed how quickly Venus expression was lost in WT-Venus-PR8 and the relationship between Venus deletion and virus titer. We passaged the viruses in MDCK cells at a multiplicity of infection (MOI) of 0.001 and measured the proportion of Venus-positive plaques (Fig. 1B). We confirmed that the expression of Venus was lost immediately in WT-Venus-PR8, whereas all plaques of Venus-PR8-PB2-E712D showed Venus expression after four passages. Although WT-Venus-PR8 showed a lower titer than Venus-PR8-PB2-E712D in MDCK cells, as described previously (8), the virus titer increased during virus passages as the proportion of Venus-positive plaques decreased (Fig. 1C). This result suggests that the loss of the Venus gene in the mutated WT-Venus-PR8 restored the replicative efficiency of the virus.

### The PB2-E712D mutation does not cause an appreciable change in polymerase fidelity.

**FIG 2:**
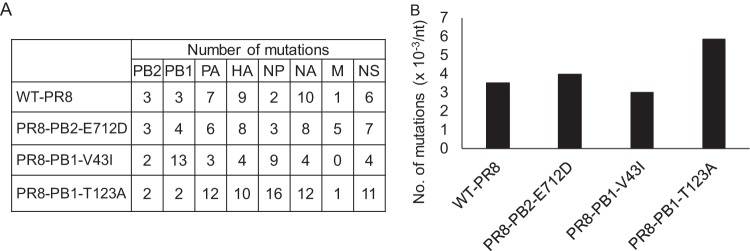
Effect of PB2-E712D on the mutation rate. (A) Each virus was passaged five times in MDCK cells, and the mutations introduced into each segment during the passages were counted. (B) The mutation number per nucleotide in each segment was calculated, and the mean values for all eight segments are shown.

We hypothesized that the PB2-E712D mutation increases viral polymerase fidelity in order to retain the inserted Venus gene during passages. To test this hypothesis, we generated WT-PR8, as well as PR8-PB2-E712D, which possesses aspartic acid at position 712 of PB2 and therefore differs from WT-PR8 by only this amino acid, by reverse genetics and compared their mutation rates. Here, we used viruses that did not contain the Venus gene to make it easier to measure the mutation rates. PR8-PB1-V43I, which has been reported to be a high-fidelity mutant virus (10, 11), and PR8-PB1-T123A, which has been reported to be a low-fidelity mutant virus (12), were also generated by reverse genetics and used as controls. To estimate the mutation rates, we passaged these viruses in MDCK cells at an MOI of 0.001 and performed deep sequencing of the entire genome; the sequencing data for the five-times passaged viruses were compared to those of viruses before passaging. We counted the number of nucleotide changes in the five-times passaged viruses that were not present before passaging. The number of mutations introduced during the five passages are shown in Fig. 2A by segment. Also, the number of mutations per nucleotide was calculated for normalization, and the mean values for all eight segments were compared (Fig. 2B). We confirmed that PR8-PB1-V43I, the high-fidelity control, had fewer mutations, and that PR8-PB1-T123A, the low-fidelity control, had more mutations than WT-PR8. Although PR8-PB1-V43I had fewer mutations than WT-PR8, the difference between PR8-PB1-V43I and WT-PR8 was small. Since a previous report suggested that PB1-V43I does not alter the mutation rate (12), the influence of PB1-V43I on the mutation rate might be dependent on the virus strain or experimental conditions. Moreover, there was no clear difference in mutation number between WT-PR8 and PR8-PB2-E712D. Although the possibility that the PB2-E712D mutation affects virus polymerase fidelity cannot be excluded, the effect does not seem to be large enough to cause an appreciable difference in Venus stability. Therefore, this result suggests that the stability of the Venus gene is not influenced by the fidelity of the virus polymerase.

### Transcription/replication of the modified RNA segment is impaired in WT-Venus-PR8.

**FIG 3:**
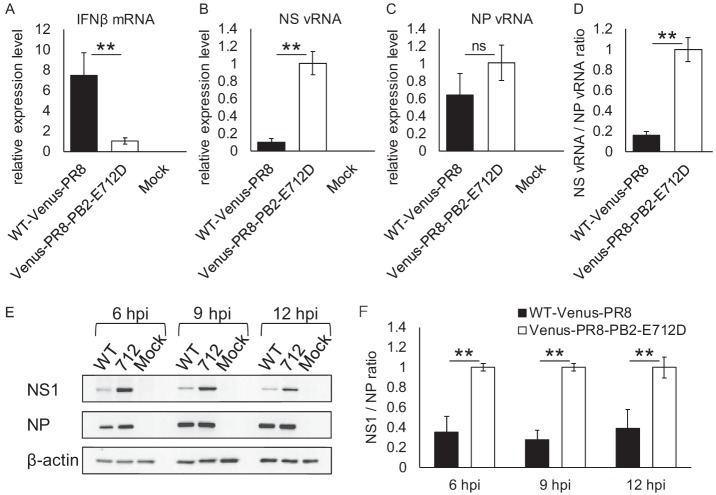
RNA and protein expression in infected cells. (A to C) MDCK cells were infected with each virus at an MOI of 1. The relative expression levels of IFN-β mRNA (A), NS vRNA (B), and NP vRNA (C) were determined by quantitative real-time PCR at 9 h postinfection. (D) The NS vRNA/NP vRNA ratio was calculated. (E) MDCK cells were infected with WT-Venus-PR8 (WT) or Venus-PR8-PB2-E712D (712) at an MOI of 1. Cells were lysed at the indicated time points, and the expression of NS1, NP, and β-actin was detected by Western blotting. (F) NS1/NP ratios were determined based on the band intensity of the Western blotting. Means ± the standard deviations of triplicate experiments, taking each value in Venus-PR8-PB2-E712D-infected cells as 1, are shown in panels A, B, C, D, and F. **, *P* < 0.01; ns, not significant (Student *t* test); hpi, hours postinfection.

Some reports suggest that recombinant viruses containing a foreign gene insertion in their NS segment can propagate more efficiently in interferon (IFN)-deficient Vero cells than in IFN-competent cells such as MDCK cells (4, 13, 14). Therefore, we quantified the expression levels of IFN-β in virus-infected cells by using quantitative real-time PCR. MDCK cells were infected with WT-Venus-PR8 or Venus-PR8-PB2-E712D at an MOI of 1 or mock infected with medium only, and the relative expression levels of IFN-β in infected cells were quantified at 9 h postinfection. WT-Venus-PR8 induced a higher level of IFN-β expression than did Venus-PR8-PB2-E712D (Fig. 3A). This result suggests that WT-Venus-PR8 does not efficiently inhibit IFN-β expression. Given that NS1 plays a key role in suppressing IFN expression and IFN-mediated antiviral responses in the host (15, 16), we next quantified the NS vRNA in infected cells by using influenza virus strand-specific real-time PCR (17, 18). The amount of NS vRNA in WT-Venus-PR8-infected cells was 90% lower than that in Venus-PR8-PB2-E712D-infected cells (Fig. 3B), whereas there was no significant difference in their NP vRNA expression levels (Fig. 3C). The NS vRNA/NP vRNA ratio in WT-Venus-PR8-infected cells was 80% lower than that in Venus-PR8-PB2-E712D-infected cells (Fig. 3D). This result suggests that the transcription/replication of the NS segment is specifically impaired in WT-Venus-PR8. The sequences of all of the plasmids used to generate viruses by reverse genetics were confirmed before use, and the NS segments of WT-Venus-PR8 and Venus-PR8-PB2-E712D were derived from the same NS-Venus plasmid. Therefore, it is unlikely that either WT-Venus-PR8 or Venus-PR8-PB2-E712D has a mutated promoter sequence in its NS segment. Accordingly, the difference in the transcription/replication efficiency of the NS segment was likely caused by the PB2-E712D. Moreover, we confirmed the expression level of the NS1 protein by Western blotting (Fig. 3E). Due to the reduced transcription/replication efficiency of the NS segment, the expression level of the NS1 protein in WT-Venus-PR8-infected cells was much lower than that in Venus-PR8-PB2-E712D-infected cells. In contrast, the expression level of NP was almost the same. The NS1/NP ratio, quantified based on the band intensity, was significantly reduced in WT-Venus-PR8-infected cells (Fig. 3F). It therefore appears that the low level of NS1 expression leads to the high expression of IFN-β in WT-Venus-PR8-infected cells. Furthermore, the high expression of IFN-β causes attenuation of WT-Venus-PR8, although it is possible that other factors are involved.

### The inserted Venus gene is deleted via internal deletion.

**FIG 4:**
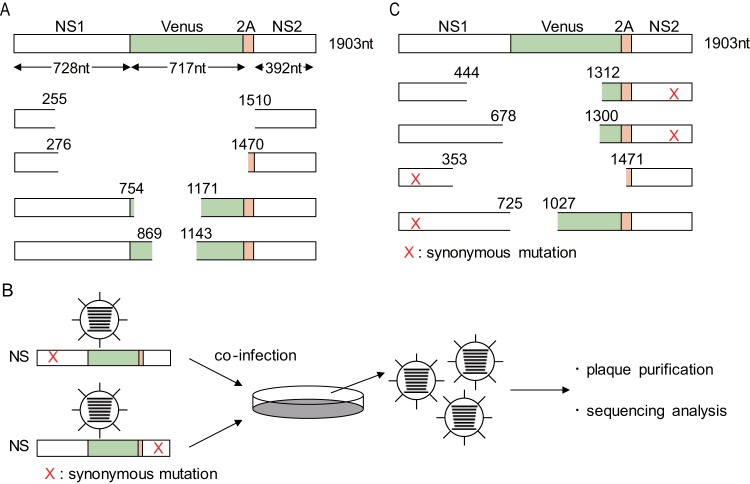
Internal deletions occurred in the NS segment of WT-Venus-PR8. (A) Schematic sequence of the NS segment in WT-Venus-PR8 viruses that lost Venus expression after serial passages in MDCK cells. Selected examples are shown. (B) The procedure for the coinfection experiment is illustrated. The synonymous mutation was introduced into the 3′ or 5′ region of the NS segment of WT-Venus-PR8. The viruses were then used to coinfect MDCK cells. Viruses not expressing Venus were plaque purified, and the sequences of their NS segments were analyzed. (C) Examples of the sequences of the NS segment of Venus-negative viruses obtained after coinfection experiments. The red “X” indicates an introduced synonymous mutation.

To explore how deletion of the Venus gene occurs, we determined the sequence of the NS segment in WT-Venus-PR8 that lost Venus expression after serial passages. We performed plaque assays using three independently passaged WT-Venus-PR8 virus stocks and found that the majority of the plaques were Venus negative. We then sequenced more than five plaques from each stock and found that one or two deletion patterns in each virus stock. We found that large deletions occurred in the NS segment and most of the Venus sequence was lost (Fig. 4A). However, we could not identify any specific patterns with regard to the deletion, such as the number of nucleotide deletions, the site of the deletion(s), or specific sequences at which deletions occurred. We hypothesized that the large deletion resulted from internal deletion caused by polymerase jumping, which is a known mechanism of defective interfering viral RNA production (19, 20), or by gene recombination, which plays an important role in RNA virus adaptation through rearrangement of the virus genome (21–24). We introduced synonymous mutations into the 3′ or 5′ region of the NS segment of WT-Venus-PR8 (Fig. 4B) and then infected MDCK cells at an MOI of 0.001 or 5 with each of these mutant viruses; supernatants were collected at 2 days or 8 h postinfection, respectively. The supernatant was then incubated with MDCK cells. Venus-negative plaques were picked up and amplified in MDCK cells, and then we determined the NS segment sequence in the viruses that lost Venus expression. We found that the truncated NS segments had a synonymous mutation on only one side (Fig. 4C). We found no viruses that had NS segments with synonymous mutations on both sides or without a synonymous mutation in both low- and high-MOI coinfections. This result indicates that the large deletion in the NS segment resulted from internal deletion in each NS segment and not gene recombination between NS segments.

### Additional mutations stabilize the Venus gene.

**FIG 5:**
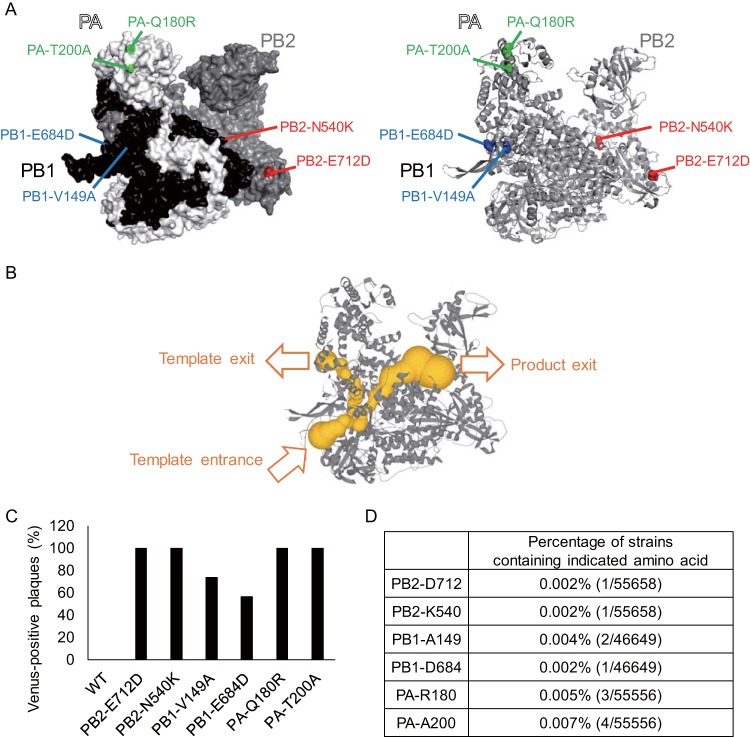
Additional mutations that stabilizes the Venus gene inserted into the NS segment. (A) Identified amino acid mutations were mapped onto the influenza polymerase complex (PDB ID 4WSB). (B) Polymerase internal tunnels (shown as yellow tubes). The vRNA promoter binds to the polymerase, and the template vRNA enters the polymerase complex. The template vRNA passes through the active site, where RNA synthesis occurs, and then leaves via the template exit. The RNA products synthesized at the active site, leave via the product exit. (C) Each mutant Venus-PR8 virus was passaged four times in MDCK cells, and the proportion of Venus-expressing plaques after passaging was determined in MDCK cells by using fluorescence microscopy. (D) Percentages of influenza A virus strains containing mutations that stabilize the Venus gene in Venus-PR8 (i.e., the number of strains containing the indicated amino acid/total number of strains available in the Influenza Research Database).

To further understand the mechanisms of the Venus deletion and stabilization, we attempted to obtain additional mutations in the polymerase complex that stabilize the Venus gene. MDCK cells were infected with WT-Venus-PR8 at an MOI of 0.001, and then Venus-positive plaques were picked and amplified in MDCK cells. After consecutive passaging and plaque purification of Venus-positive viruses, we obtained mutants that stably expressed enhanced Venus fluorescence. Sequence analysis revealed that mutations were introduced into the polymerase genes PB2, PB1, and PA of each mutant (Fig. 5A). PA-180 and PA-200 are located on the surface of the polymerase complex, as is PB2-712, whereas PB2-540, PB1-149, and PB1-684 are located inside the complex. PA-180 and PA-200 are located in the endonuclease domain, PB1-149 and PB1-684 are located near the exit of the RNA template, and PB2-540 is located near the exit of newly synthesized RNA products (Fig. 5B), while the function of the region around PB2-712 has remained unclear (25–27). To determine whether these mutations contribute to the stabilization of the Venus gene, we generated mutant viruses containing each of the mutations by using reverse genetics and measured the Venus retention ratio after four passages in MDCK cells (Fig. 5C). The mutant viruses showed enhanced Venus stability compared to WT-Venus-PR8, indicating that these amino acids play important roles in the stabilization of the Venus gene. Although further analysis is needed to clarify how these amino acids contribute to the stability of the Venus gene, considering that PB2-540, PB1-149, and PB1-684 are located near the polymerase internal tunnels (25–27) that the template and product go through during the transcription/replication reaction, these amino acids may affect the binding stability of the RNA template, product, and polymerase complex. Moreover, when we examined whether these mutations were found in previously isolated influenza A viruses in the Influenza Research Database (Fig. 5D), we found that these amino acids are extremely rare, suggesting that they are not evolutionarily beneficial.

## DISCUSSION

Recombinant influenza viruses expressing foreign genes would be useful tools; however, long insertions in virus genomes are often unstable and cause attenuation of the recombinant viruses. We previously found that amino acids in the influenza virus polymerase complex play crucial roles in the stabilization of foreign gene insertions; the Venus gene inserted into the NS segment was stabilized by PB2-E712D in an H1N1 virus (7, 8) and by PB2-V25A and PA-R443K in an H5N1 virus (9). However, the mechanisms by which these amino acids contribute to the stabilization remained unclear. In the present study, we explored the mechanism of PB2-E712D-induced stabilization of the Venus gene inserted into the NS segment of an H1N1 virus. We found that the transcription/replication efficiency of the modified segment was significantly reduced in WT-Venus-PR8 compared to Venus-PR8-PB2-E712D. This finding suggests that the PB2-E712D mutation stabilizes the inserted foreign gene due to the enhanced transcription/replication efficiency of the modified RNA segment. In contrast, the transcription/replication efficiency of segments that do not contain additional sequences is not changed in the presence or absence of the PB2-E712D mutation. Moreover, we previously showed that polymerase activity is reduced, not enhanced, by the PB2-E712D mutation in a minireplicon assay (8). These results indicate that the alteration of the transcription/replication efficiency caused by PB2-E712D is specific to modified RNA segments. The insertion of foreign genes appears to impair the transcription/replication of the modified segments, and the polymerase overcomes this impairment in the presence of the PB2-E712D mutation.

In WT-Venus-PR8, in which the Venus gene is inserted into the NS segment, the transcription/replication efficiency of this segment is significantly reduced. As a result, the expression of the NS1 protein is also reduced. Since NS1 plays an important role in inhibiting IFN-mediated antiviral responses (15, 16), WT-Venus-PR8 cannot inhibit IFN-β expression efficiently, which may lead to virus attenuation. The viral titer of WT-Venus-PR8 increases during serial passages in MDCK cells as the virus loses Venus expression (Fig. 1B and C), suggesting that mutated WT-Venus-PR8 that does not contain the Venus gene propagates more efficiently than the original WT-Venus-PR8. Therefore, it is likely that the immediate loss of Venus expression in WT-Venus-PR8 results from the selection of variants without the Venus gene during serial passaging. Venus-PR8-PB2-E712D restores the transcription/replication efficiency of the NS segment, leading to efficient virus replication. Therefore, viruses expressing Venus are not purged by selective pressure in the presence of the PB2-E712D mutation, which enables Venus-PR8-PB2-E712D to stably maintain the inserted Venus gene.

How is the transcription/replication efficiency reduced on modified RNA segments specifically, and how is it enhanced by the PB2-E712D mutation? The RNA secondary structure and the binding affinity between the polymerase complex and the RNA templates likely hold the answer to these questions. Insertion of a foreign gene must change the RNA secondary structure, and transcription/replication by the viral polymerase complex may be negatively influenced by this unusual RNA secondary structure. Although we do not conclusively know how the PB2-E712D mutation overcomes the impairment of transcription/replication, one possible explanation is that the binding affinity between the polymerase complex and the RNA templates is increased.

Our sequence analysis of WT-Venus-PR8 that lost Venus expression, coupled with the coinfection experiments (Fig. 4B and C), suggested that the inserted sequence is deleted due to an internal deletion. Internal deletions often occur during influenza virus replication cycles regardless of the presence of a foreign gene insertion and have been reported to play roles in virus adaptation (28–30) and the generation of defective interfering viral RNA (19, 20). Internal deletion is believed to be caused by polymerase complex dissociation from RNA templates during transcription/replication (20, 31–33). Amino acid mutations in the polymerase complex affect the frequency of occurrence of internal deletions (34–37). PB2-E712D may also be involved in the occurrence of internal deletions. Therefore, the stabilization of the Venus gene in Venus-PR8-PB2-E712D may be caused not only by the enhancement of the transcription/replication on the modified segment but also by the reduced frequency of internal deletions.

We also identified additional mutations in the influenza virus polymerase complex that stabilize the inserted Venus gene, which may help us to further understand the stabilization mechanisms based on the positions of these mutations in the viral polymerase complex. Some of the identified amino acids are located near the polymerase internal tunnels, which are near the RNA template or newly synthesized RNA product during the transcription/replication reactions (25–27). These amino acids might directly affect the binding affinity between the polymerase complex, template, and product. A previous report, which showed that PB2 amino acids located at the template exit channel are involved in the formation of short aberrant RNAs (37), supports the possibility that amino acids near the polymerase internal tunnels affect the binding affinity between the polymerase complex, template, and product. However, PA-180 and PA-200, which are located at the endonuclease domain, are not near the polymerase internal tunnels, which is also true for PB2-712. Therefore, these amino acids may affect the binding affinity indirectly, or there may be other mechanisms involved in the stabilization of the Venus gene. These mutations could be used to establish recombinant influenza viruses expressing a foreign gene. However, these amino acids may not necessarily cause the stabilization of a foreign gene in all influenza virus strains, since PB2-V25A, which stabilizes the Venus gene in Venus-H5N1, had a negative effect on virus replication in Venus-PR8 and did not cause Venus stabilization (our unpublished data).

Although the identified amino acids seem to enhance the genetic stability of virus genomes, they have been rarely found in virus isolates (Fig. 5D). It seems likely that mutations that support the maintenance of inserted sequences are not evolutionarily beneficial to the virus. Insertions of additional sequences into virus genomes are often deleterious for virus replication. These mutations are probably rare in virus populations to avoid the accumulation of deleterious insertions. Viruses may purge deleterious insertions by reducing the transcription/replication efficiency of RNA segments that contain insertions that form abnormal secondary structures. In conclusion, although the amino acid mutations we identified in this study are useful for generating recombinant viruses, they do not seem to be beneficial to the virus in nature in the long run.

## MATERIALS AND METHODS

### Cells and viruses.

Madin-Darby canine kidney (MDCK) cells were cultured in minimal essential medium (Gibco) with 5% newborn calf serum at 37°C in 5% CO_2_. Human embryonic kidney 293T (HEK293T) cells were cultured in Dulbecco's modified Eagle medium supplemented with 10% fetal calf serum. WT-Venus-PR8 and Venus-PR8 mutants with NS segments encoding the Venus fluorescent protein (7) were generated by using reverse genetics (1) and propagated in MDCK cells at 37°C.

### Venus stability.

MDCK cells were infected with WT-Venus-PR8 or each Venus-PR8 mutant at an MOI of 0.001. The supernatants were collected at 48 h postinfection and titrated by using plaque assays in MDCK cells. Obtained viruses were similarly passaged four times. The proportion of Venus-expressing plaques in virus stocks from different passages was determined in MDCK cells by observing more than 65 plaques in each virus stock using fluorescence microscopy. To exclude false-positive plaques, Venus-negative plaques were picked up, amplified in MDCK cells, and reassessed for Venus expression.

### Deep sequencing analysis.

WT-PR8, PR8-PB2-E712D, PR8-PB1-V43I (10, 11), and PR8-PB1-T123A (12) were generated by reverse genetics (1), and MDCK cells were infected at an MOI of 0.001. The supernatants were collected at 48 h postinfection and titrated by using plaque assays in MDCK cells. The obtained viruses were passaged five times in the same way. Virus RNA was extracted from viruses before passaging and from viruses passaged five times by using a QIAamp viral RNA minikit (Qiagen). Reverse transcription-PCR (RT-PCR) was performed by using a Superscript III high-fidelity RT-PCR kit (Invitrogen). DNA amplicons were purified by 0.45× of Agencourt AMpure XP magnetic beads (Beckman Coulter), and 1 ng was used for barcoded library preparation with a Nextera XT DNA kit (Illumina). After bead-based normalization (Illumina), libraries were sequenced on the MiSeq platform in a paired-end run using the MiSeq v2, 300 cycle reagent kit (Illumina). The raw sequence reads were analyzed by using the ViVan pipeline (38). Here, a cutoff of 1% as the minimum frequency was used. Moreover, we defined an empirical cutoff for the minimum read coverage: for a variant with 1% frequency, at least 1,000 reads should cover that region. Likewise, for a variant with 0.1% frequency, 10,000 reads should cover that region. Namely, if the coverage was <1,000/(frequency), the variant was removed. The sequencing data of the five-times passaged viruses were compared to those of the viruses before passaging. The number of nucleotide mutations that were not observed before passaging but observed only after passaging was counted. The number of mutations per nucleotide was calculated for each segment and the mean values for all eight segments in each virus were compared.

### Quantitative real-time PCR.

MDCK cells were infected with WT-Venus-PR8 or Venus-PR8-PB2-E712D at an MOI of 1 or mock infected with medium. The total RNA was extracted from cells at 9 h postinfection by using an RNeasy minikit (Qiagen). Quantification of RNA was performed as described previously (17). The primers for IFN-β, NS vRNA, NP vRNA, and β-actin were described previously (17, 18, 39). Data were analyzed with the 2^–ΔΔ^*^CT^* method (40) and normalized to the expression of β-actin mRNA.

### Western blotting.

MDCK cells were infected with each virus at an MOI of 1 or mock infected with medium. Cells were lysed at the indicated time points with Tris-glycine SDS sample buffer (Invitrogen). The cell lysates were sonicated, heated for 10 min at 95°C, and then subjected to SDS-PAGE. SDS-PAGE was performed on Any kD Mini-PROTEAN TGX precast protein gels (Bio-Rad). Proteins on SDS-PAGE gels were transferred to a polyvinylidene fluoride membrane (Millipore) and detected by using the indicated primary antibodies (rabbit anti-NS1 [GeneTex], mouse anti-Aichi NP [2S 347/4], mouse anti-β-actin [Sigma-Aldrich]), followed by secondary antibodies (sheep horseradish peroxidase [HRP]-conjugated anti-mouse IgG [GE Healthcare] or donkey HRP-conjugated anti-rabbit IgG [GE Healthcare]). Signals of specific proteins were detected by using ECL Prime Western blotting detection reagent (GE Healthcare). Images were captured with a ChemiDoc Touch imaging system (Bio-Rad) and quantified by using Image Lab software (Bio-Rad).

### Coinfection analysis.

Three nucleotides in the 3′ or 5′ region, which does not overlap the packaging signal sequence (41) of the NS segment of WT-Venus-PR8, were substituted synonymously. These modified viruses were used to coinfect MDCK cells at an MOI of 0.001 each or an MOI of 5 each. The supernatant was collected at 2 days or 8 h postinfection, respectively, and infected to MDCK cells. Venus-negative plaques were picked up and amplified in MDCK cells, and then the sequences of the NS segments in the obtained viruses were analyzed.

### Identification of additional mutations that stabilize the Venus gene.

WT-Venus-PR8 was infected to MDCK cells at an MOI of 0.001. The supernatant was collected at 2 days postinfection and infected to MDCK cells. Next, Venus-positive plaques were picked up and amplified in MDCK cells repeatedly until mutants stably expressing Venus fluorescence were obtained. The sequences of the mutants were analyzed to identify amino acid mutations in PB2, PB1, and PA. To determine whether these mutations contributed to the stability of the Venus gene, mutants containing each of the identified amino acid mutations were generated by reverse genetics (1), and the Venus stability of each mutant was examined as described above. Confirmed amino acid positions were plotted on the crystal structure of the influenza virus polymerase complex (PDB ID 4WSB) by using the PyMOL molecular graphics system. In Fig. 5B, the polymerase internal tunnels were visualized by using the MOLEonline web interface (42), and the information was deposited in ChannelsDB (43). The percentage of strains that contained the identified amino acid was determined by using the “Sequencing Feature Variant Type” tool in the Influenza Research Database (44, 45).

### Statistical analysis.

Statistically significant differences between WT-Venus-PR8 and Venus-PR8-PB2-E712D were assessed by using a two-tailed unpaired Student *t* test. A *P* value of <0.05 was considered significantly different.
